# Medical ethics as practiced by students, nurses and faculty members in Shiraz University of Medical Sciences

**Published:** 2015-01

**Authors:** LEILA BAZRAFCAN, PARISA NABEIEI, NASRIN SHOKRPOUR, NEDA MOADAB

**Affiliations:** 1 Quality improvement in clinical teaching Research Center, Shiraz Education Center, Faculty of Medical Education, Shiraz University of Medical Sciences, Shiraz, Iran;; 2 English Department, Para Medical School, Shiraz University of Medical Sciences, Shiraz, Iran;; 3 Shiraz University of Medical Sciences, Shiraz, Iran;

**Keywords:** Ethics, Medical ethics, Student, Nurse, Faculty

## Abstract

**Introduction: **Assuming any social role has obligations and fulfilling the related responsibilities has ethical aspects that must be addressed carefully. Each role requires extensive training, which usually takes place in university institutions**. **Ethics is applied in at least three academic areas, including: a) in education of students' personal growth, b) in patient care, and c) in university communion in population-based health care. Given the importance of this issue in the moral domain, this study examines the correlation among the students, nurses and teacher's opinions regarding principles of medical ethics at Shiraz University of Medical Sciences.

**Methods:** This is a descriptive-analytic and cross-sectional study conducted in 2010. The participants of this research consisted of all medical students, nurses in public hospitals, and faculty members in Shiraz University of Medical Sciences. For validity evaluation, the expert panel method and for reliability evaluation, test-retest method was used.

**Results:** Based on the medical ethics’ scores in these three groups, there was a significant relationship between the mean scores of student-nurses and employed nurses, but there was no significant relationship between those of student-faculties. Also the mean score of the students was the highest in medical ethics.

**Conclusion:** In this study, we presented a list of virtues and moral characteristics of medical staff and found out the method of practicing medical ethics in everyday life of students to improve the moral reasoning of teachers, nurses and students. Moreover, medical ethics, with the presentation of specific criteria for ethical behavior in various domains of human life, especially in dealing with patients, can help practice ethical values in the medical community.

## Introduction

Assuming any role in the society has obligations and fulfilling these responsibilities entails ethical aspects that must be addressed carefully. Each role requires extensive training, which is usually offered by university institutions. This training includes complex components of thought, such as acquisition of a lot of theoretical and practical knowledge. However, any role provides significant services for the community. So, each role can be called a profession which has specific responsibilities. Basically, owners of professions have certain experiences and backgrounds that should be applied by the community in order to receive their services responsively. Medical knowledge brings about so much power and authority to profession owners, medical students and teachers and if it is used without observing the ethical aspects, there will be the risk of improper practice (1).

Ethics has a specific place at least in three academic areas: 1) in education of students' personal growth, 2) in patient care, 3) in university faculties in population-based health care. In education, ethics must not be matched by a group of facts that should be considered as one of ethical issues of the curriculum; on the contrary, the intention is to encourage students to think about practicing of ethical issues in medicine carefully (2).

In patient care, nurses and students can continuously exchange their views on the moral content of problems. For example, is using the body organs of a person who is brain dead in order to save another person’s life, notwithstanding the worth of saving a second person, ethical? Other ethical issues may occur in the population-based health care. For example, as to the approval of the community before investigation, many issues are raised which are ethically controversial (1, 2).

Due to the importance of ethics in the medical field, in this study we first define the term ethics in the medical profession. Medical ethics is an analytical activity in which thoughts, ideas, commitments, attitudes, feelings, arguments and discussions on various aspects of ethical decision making in the medical profession are studied carefully and critically and the necessary instructions are provided. Medical ethics decisions in the field of medicine are specific issues, values and generally dos and don'ts (medical ethics, including a brief medical history, cultural affairs and the Ministry of Health and Medical Education, 1991) (3).

Despite the long history of this science, medical ethics courses for medical students have been offered for about 4 years as formal courses of medical schools around the world. In 1993, General Medical Committee (GMC) posed recommendations regarding the medical students' education, leading to an increase in motivation and driving force for improving principles of medical ethics education, so medical ethics training became a part of the core curriculum in England medical schools. Thus General Medical Committee and internal medicine board prepared a set of skills and competencies for physicians to be included in the curriculum of medical schools and the training of medical students. This provided a basis for evaluating medical ethics regarding the profession owners in different medical fields (4).

But does the training the lecturers on medical ethics in the universities of medical sciences guarantee appropriate and timely implementation of ethics by graduates? And is the students’ practice after the formal and academic training on medical ethics by authorities and in health care environments, similar? In fact, this is a subject that has preoccupied many experts in medical ethics, and the main goal of this research is to investigate this issue.

Given the importance of this issue in the moral domain, this study examines the correlation among students, nurses and teacher's opinions regarding principles of medical ethics at Shiraz University of Medical Sciences.

In Indiana University (2000), a checklist with 22 items on moral characteristics and traits was used to assess the behavioral and vocational errors. Four students were trained to observe these behaviors. The results showed some deficiencies in professional behavior of students (5).

Glascow University evaluated the new student-centered problem-based ethics program on 238 first year medical students using qualitative multi-method approach. Evaluation tools were open questionnaires, focus groups and instructors checklist; 30 clinical teachers functioned as facilitators of learning. Male and female students were selected using phase sampling to participate in the focus groups. The results showed that training in small groups for teachers and students was very acceptable (6).

Every year at the University of Florida, classmates and peer evaluation about ethical values are done routinely; the students are asked to introduce 7 classmates who are better in developing clinical competencies and personal relationships (7).

Bazrafkan et al. (2001) did a cross-sectional research about clinical competencies of medical students in relation to the clinically common illnesses in Shiraz University of Medical Sciences. The study population consisted of 120 interns who were to be graduated. This study was done through implementation and evaluation of twenty OSCE stations about outpatients' disease. The results of this study showed that students do not have the necessary sufficient clinical competence or ability as to human relationships, i.e. even to gain score; they failed to act properly (8).

## Methods

This research is a descriptive- analytic and cross-sectional study conducted in 2010. The participants of this research included all medical students, nurses in public hospitals, and faculties in Shiraz University of Medical Sciences. The sample size of this study was calculated by Quota sampling and randomization as follows: 120 medical students, 160 nurses of public hospitals and 100 faculty members in medical school.

Data were gathered using a researcher-made questionnaire. The questionnaires were completed by participants. The questionnaire had 2 parts; the first part included 3 questions on the demographic characteristics (age, sex and field of study) and the second part contained 30 multiple choice questions in 5 dimensions of responsibility, virtue ethics, conflict resolution, and respect and altruism. Each dimension had 6 questions. The questions in this questionnaire were based on the GMC list for professional competence by the researcher.

At the top page of the questionnaire, it was explained that personal information is completely secret, and names and families are not mentioned. People were asked to answer the questions honestly in order to use the collected information in designing educational programs to enhance medical ethics level. 

Likert scale was used for scoring including (always, most of the time, sometimes, rarely, never) and their allocated scores were 1 to 4, respectively. The minimum obtained grade was 33 and the maximum 165.

For validity evaluation, expert panel method was used (5 members of specific board of medical ethics of Shiraz University of Medical Sciences) and for reliability evaluation, test-retest method was used. The adjusted questionnaire was distributed among 18 faculty members of Shiraz University of Medical Sciences, twice during 10 days. The Cronbach coefficient was evaluated using SPSS, version14, software and this coefficient for the final questionnaire was 83%. Then, the questionnaires were distributed among the target groups and correctly completed questionnaires were collected.

For data processing, SPSS software, version14 was used and data were analyzed using t-test and ANOVA. In this research, the relationship between medical ethics and demographic information was determined and shown with descriptive and analytic frequency Tables. 

## Results

380 faculty members, students, and nurses of university hospitals participated in this study. Of them, 142 (37%) were 30-20 years old, 148 (39%) were 40-30 years old and 90 (24%) were aged 50-40 years. Of these, 258 (68%) were women and 122 men (32%). In addition, 136 individuals (36%) were married and 244 (64%) single.

About the principles of professional ethics from the perspective of the participants as to responsibility, 63% of people stated that they observed the standards of professional ethics. 5% of the participants had poor performance.  In the dimension of moral virtues, 78% of the participants stated that their performance in this area was desirable and only 2% of them believed that their performance was undesirable (Table 1).

**Table 1:** Distribution and percentage of the quality of participant’s ethical performance compared with the professional ethics standards in the studied aspects

**Dimension** **Quality of** **Performance**	**Responsibility** **N (%)**	**Improving the quality of patient care** **N (%)**	**Respect to patient** **N (%)**	**Conflict Resolution** **N (%)**	**Altruism** **N (%)**
**Poor performance**	20 (5)	53 (14)	48 (12)	36 (9)	8 (2)
**Relatively good performance**	120 (32)	248 (65)	135 (36)	182 (48)	76 (20)
**Optimal Performance**	240 (63)	79 (21)	197 (52)	162 (43)	296 (78)

The mean score and standard deviation with respect to the patients dimension in the students’ group were the highest (26.05±6.37) and conflicts resolution dimension in the nurses’ group was the lowest (10.18±2.76). In addition, the mean score of the conflict resolution dimension (12.02±2.92) was the lowest score and in the dimension of respect to patient (21.95±5.92) it was the highest score (Table 2).

**Table 2:** Mean scores of the participants in the study

** Dimension** **Participation**	**Responsibility**	**Virtue ethics**	**Conflict resolution**	**Respect**	**Altruism**
Students	24.01±5.15	19±4.96	14.72±3.76	26.05±6.37	19.69±4.98
Nurses	15.38±3.98	15.39±3.98	10.18±2.76	18.42±4.98	12.52±3.32
Faculty	16.32±4.12	15±3.97	10.35±2.80	19.06±4.95	13.06±3.48
Total	19±4.96	16.76±3.99	12.02±3.21	21.59±6.02	15.50±3.98

**Table 3:** Correlation between demographic variables and professional ethics dimensions and views of different groups

**Correlations between different variables**	**Spearman’s correlation coefficient**	**Pearson's correlation coefficient**	**ANOVA **
**Sex and the average scores of professional ethics**	** p=0.08 r=0.32**	**-**	**-**
**Marital status and** **mean scores of professional ethics**	** p=0.15 r=0.46**	**-**	**-**
**Age and mean scores of professional ethics**	**-**	** p=0.06 r=0.28**	**-**
**Views of Students and faculty**	**-**	**-**	**p=0.902**
**Views of faculty and nurses**	**-**	**-**	**p=0.001**
**Views of students and nurses**	**-**	**-**	**p=0.002**

The results of Pearson's test showed that there was no significant correlation between age and mean scores of professional ethics (p=0.006, r=0.280); according to the Spearman test, there was no significant correlation between gender and professional ethics mean scores and marital status and mean scores of morality (p=0.008, r=0.320; p=0.250, r=0.460). Also results of ANOVA test between the three groups showed that there was no significant relationship between comments of faculty members and students’ groups (p=0.902), but there were significant relationships between the comments of faculty-nurse and student nurses’ groups, (p=0.001, p=0.002) (Table 3).

## Discussion

As to the principles of professional ethics from the perspective of the participants in the dimension of responsibility, 63% of people stated that they observed the standards of professional ethics. 5% of the participants had poor performance .in the dimension of moral virtues. 78% of people stated that their performance in this area was desirable and only 2% of them believed that their performance was undesirable. In a research study (2012), 24% of the participants stated that their performance was desirable but the performance of 4% of them was undesirable (6).

In the study of Sokhanvar et al., the application of nursing ethics was reported poor (23.9%) (7). In the current research, among ethical issues, dimension of respecting the patient obtained the highest mean score in the students group (26.05±6.37) and dimension of conflict resolution had the lowest score in the nurse group (10.18±2.76).

Dehghani (2012) stated that dimension of responsibility and conflict resolution had the highest and the lowest mean scores so that the nurses stated that they showed the lowest response against non-compliance of professional ethics by their colleagues and action recording faithfully for the patient and reports of error (6).

In a research conducted by Mardani et al., 95.6% of the participants stated that staff errors should be reported, and this is the most important factor of respecting the patient rights, but there are a number of factors including legal issues of managers’ inadequate feedback about reporting of errors and tensions with colleagues that are considered the most important obstacles (8).

Observing the patient's rights in our study population was considered desirable. In a study conducted by Haghdoost et al. at Kerman University of Medical Sciences, there were significant differences between the perspective and perceptions of faculty members and students;  this finding was not in the same line with the results of this study (9).

According to the findings, there was no significant relationship between age and marital status with quality of professional ethics. This finding was consistent with the findings of Dehghani (2012) (6). Also, Sokhanvar et al. reported that there was a significant relationship between gender and the use of ethical principles; the results were not consistent with those of this study (7).

Since in this study we aimed to determine the correlation among the views of students, nurses and faculty members of Shiraz University of Medical Sciences in the field of professional ethics; the results indicated a significant correlation between the views of all three groups (faculty members, students and student nurses) in the field of medical ethics.

Several studies in different parts of the country and the world have investigated this issue. For example, in order to understand the professional ethics, in Canada a study was performed on 108 medical students who had studied for one year at the University of Toronto. The students were asked how often they had been in clinical situations and forced to do immoral behavior. Nearly half of the students (47 percent) argued that this was too much or it sometimes happened.When they were asked how often they had witnessed unethical behavior of faculties, 61 percent of them stated that this situation had happened a lot for them (6, 10).

As observed in this study, the doctors at work, sometimes in the presence of students, ignored ethics; this could be due to unfavorable climate, current affairs, and hasty or inappropriate training. Since university faculty members affect the environment and students, they should be equipped with moral literacy, be familiar with its principles and be adorned with moral virtues (11).

So the moral education of physicians and nurses in the hospital environment is very important. As seen in this study, the views of these two groups in the workplace were inconsistent. Some studies have focused on the role of faculty members as a moral figure during the course (12).

Students were asked how often they had been forced to do immoral behavior in clinical situation. Half of them (47%) stated that this was seen frequently or sometimes happened for them. When they were asked how often they had witnessed unethical behavior of faculties, 61 percent of them said that this happened for them a lot (6).

Also, as observed in this study, the physicians at workplace, sometimes in the presence of students ignored ethics that this could be due to unfavorable climate, current affairs, and hasty or improper training. Since faculty members affect the students and their environment, they should be equipped with moral literacy and be familiar with its principles and moral virtues (11).

On the other hand, the ethical dimensions which consisted of five dimensions (responsibility, personality traits, problem solving, respect, dignity and altruism) indicate that the mean scores in all dimensions were more than those of the other groups.This means that students' mean scores were higher in all aspects. In other words, it can be concluded that the observance of professional ethics in the students group was more than that in the other two groups. This shows that faculty members and nurses in the workplace have focused less on medical ethics. Also, the highest mean scores were related to the honor and dignity and lowest to the conflict resolution.

In a study conducted in a general hospital in Tehran, the physicians were asked to rate the ethical issues they have encountered during their clinical practice based on the list from zero to ten points (Zero, the lowest score and 10 the highest score). The results of 34 physicians participating in this study showed that the most common ethical issues were confidentiality (with a score of 7.1 out of 10), conflict resolution, communication with colleagues, and financial relationship of patients and physicians (13); these findings were in the same line with the results of the present study.

Several training programs have been conducted at the university level.  For example,  There has been an attempt to hold medical ethics workshops at Shiraz University of Medical Sciences, making them more attractive and effective.  However, unfortunately little attention has been paid to ethical issues (14). However, most people in the medical community are familiar with many of the moral issues but in practice they do not observe them, so they usually face with two main problems:

1. They cannot properly detect the way of doing moral duties and the practice of moral judgments in their work environment
2. They cannot make a proper decision about the conflict of moral duties (15).

Wehrwein believes that teaching ethical issues is effective to raise awareness of students about ethical issues and the application of ethics in the workplace and ethical decision-making skills. The results of this research are evidence on the importance of moral education and its positive impact on improvement of nursing students' moral judgment (16).

20 lecturers in nursing in the United States that participated in the study believed that moral education had the most influence on the moral and spiritual development of students, colleges and faculties have a major role in shaping the ethical framework. Woods in his review came to the conclusion that the role of models in moral education is very important. He recommends that in order to help the students to have insight into the professional ethics, faculties should be trained (17).

## Conclusion

Agreement among different groups (students and faculty members, students and nurses) reveals the favorable results, and disagreement between groups of faculty members and nurses might be due to different types of training.

Since medical ethics is an inter-professional issue, planning and organizing various medical groups (students, faculty members and staff) is very important. In fact with the joint training of these groups, an opportunity is provided to engage in thinking about ethical issues in health system; in this way, of the discrepancy in views and opinions is removed. In order to prevent any medical error in a hospital environment, setting a monitoring system is necessary. On the other hand, appropriate training should be properly performed in medical schools and educational centers which are affiliated to universities or private academic and non-academic centers for familiarity with the existing laws and further control and surveillance.

Due to the limited number of studies in the field of professional ethics in Iran as compared to other countries, the results of the studies reveal that there is a need to more attention, action planning, education and research as to the students, teachers and nurses' professional ethics knowledge and practice. 

As obvious, professional ethics codes and components have been developing in our country for many years and students and hospital medical staff (faculty members and nurses) will respectfully practicing them. Moral concepts have positive meaning, giving rise to a good and lovely feeling in all people. Therefore, these concepts are valuable and familiar to them but in decision making in real contexts and in the presence of the patient, only the knowledge about moral issues and their value is not sufficient. 

In fact, medical ethics as viewed by the experts is a subject which requires planning and designing among different groups of medical sciences (students, teachers and nurses). The significant difference in the opinions of these groups may also be due to different types of training and lack of appropriate executive sponsorship at the university level. Therefore, teachers in different disciplines should have enough time for consultation and thinking about ethics; this sharing of the comments makes the ground to overcome differences in the views of educational groups. It is necessary for the university authorities to make decisions as to medical ethics at the health care settings so that the context for implementing these principles will be further and more efficiently provided. 

**Limitations of the study**

As in any other research, the limitations of the present study are as follows:

1. High costs for implementation of the program at the university
2. Lack of related resources in the country.
3. Problems relating to the justification of experts and practitioners about the implementation of the principles of medical ethics in health system
